# 1:2 entrainment is not a device-induced artefact, except when it is

**DOI:** 10.1016/j.brs.2024.01.010

**Published:** 2024-02-06

**Authors:** James J. Sermon, Moaad Benjaber, Benoit Duchet, Juan Anso, Maria Olaru, Philip A. Starr, Timothy Denison

**Affiliations:** a Institute of Biomedical Engineering, Department of Engineering Science, University of Oxford, Oxford, UK; b MRC Brain Network Dynamics Unit, Nuffield Department of Clinical Neurosciences, University of Oxford, Oxford, UK; c Department of Neurological Surgery and Weill Institute for Neurosciences, University of California San Francisco, San Francisco, CA, USA

Dear Editor,

Deep Brain Stimulation (DBS) is a well-established invasive therapeutic approach for Parkinson’s disease (PD). However, the mechanisms of DBS remain unclear. A transition from the established open-loop paradigm to closed-loop DBS holds promise for enhancing therapeutic outcomes and optimising device efficiency [1,2]. This transition requires identifying reliable biomarkers for the closed-loop algorithm. Recent studies suggest that biomarkers associated with harmonics of stimulation frequency may offer viable options for adaptive DBS (aDBS) and provide mechanistic insights [2,3]. Previously subharmonic biomarkers have been avoided, often dismissed as artefactual. This is partly due to there being no clear physiological/non-physiological differentiating factor when considering steady signals during short time periods. To advance sense-stimulation therapies and aDBS, it is imperative to identify artefact-free frequency bands for effective biomarker monitoring. Here, we present a framework for discerning artefacts at the half harmonic of stimulation frequency.

## Do we expect entrainment? Yes!

**Fig. 1:**
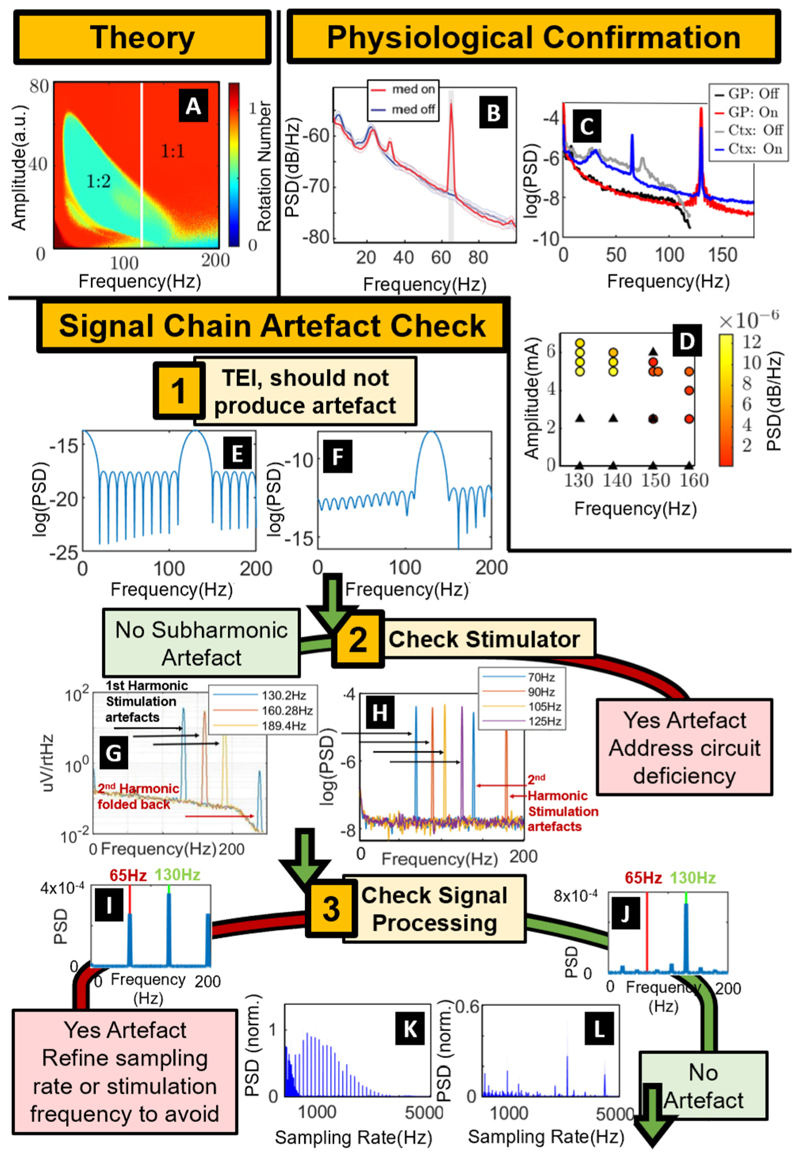
A flow diagram for addressing entrainment vs artefact. Theory: (A) Prediction of entrainment regions from a two-population Wilson-Cowan model fitted to off-stimulation data of the motor cortex, adapted from [3]. The 1:2 entrainment region (1:2 Arnold tongue) is indicated by the green region with a rotation number of 0.5 and the 1:1 entrainment region in red. Physiological Confirmation: (B) The modulation of 1:2 entrainment in different medication states with 130 Hz stimulation, adapted from [2]. (C) The modulation of 1:2 entrainment with recording location (globus pallidus (GP) and cortex (Ctx)) for both on pallidal stimulation at 130 Hz and off-stimulation conditions. (D) The modulation of 1:2 entrainment with stimulation parameters, adapted from [3], circles indicate 1:2 entrainment observed with power spectral density (PSD) corresponding to the colour. Black triangles indicate no 1:2 entrainment. Signal Chain Artefact Check: (E and F) The spectrum of the voltage outputs of a resistor capacitor circuit model (E) of a tissue-electrode interface (TEI) and a resistor network (F) TEI [7] stimulated at 130 Hz with monophasic stimulation pulses. (G and H) The outputs of a summit RC+S device [9] (G) and a DyNeuMo-2 (H) in saline tank recordings for a range of stimulation frequencies. (I and J) The PSD of 130 Hz monophasic stimulation pulse train after downsampling through a delta-sigma analogue-to-digital converter (ADC) with sampling rate 3185 Hz (I, half harmonic artefact) and 3100 Hz (J, no half harmonic artefact). (K and L) The PSD at the half harmonic (65 Hz) normalised by the PSD at stimulation frequency (130 Hz) after downsampling through a conventional ADC (K) and delta-sigma ADC (L).

It should be noted that theoretically we expect to see subharmonic entrainment to periodic perturbations in certain nonlinear systems [4]. Cortical 1:2 entrainment of finely tuned gamma (FTG) through DBS has been demonstrated to occur in a predictable manner in PD [3] (Fig. 1A). A key requirement is believed to be neuronal ensembles with a natural frequency close to harmonics of stimulation frequency (discussed in [3]). This can be seen in Fig. 1C where 1:2 entrainment is only observed in the cortex which displays FTG activity at approximately 75 Hz off-stimulation, unlike the pallidum where there is no activity around the half harmonic of stimulation. Theory can account for entrainment as a neurophysiological phenomenon but further checks are required to rule out the presence of artefacts.

## Physiological confirmation steps

Initially, it is important to treat subharmonic activity cautiously and as an artefact. However, when evidence demonstrates physiological origin we must be open to exploit the potential clinical implications. We can demonstrate physiological origin by considering how subharmonic entrainment evolves around independent perturbations to physiology or stimulation. **1) Medication dependent:** 1:2 FTG entrainment is modulated by medication and is only present in on-medication states [2,5] (Fig. 1B). **2) Sleep-wake state dependent:** Neural activity may selectively entrain at subharmonics of stimulation frequency according to sleep state [6]. In a system where stimulation and signal processing remain unchanged, for subharmonic entrainment to appear or disappear solely reflects a change in underlying neural circuitry. **3) Clinical observations:** FTG power entrained at half stimulation frequency has been shown to predict prokinetic states in PD patients, and was identified as the optimal control signal for aDBS for reducing residual motor fluctuation [2]. **4) Location Specific:** 1:2 entrainment is only observed in specific neural structures (Fig. 1C, from RCS10 as reported in [3]). The observation of 1:2 entrainment in response to pallidal stimulation is limited to cortical contacts. These contacts are not close to the stimulating electrode. This indicates that volume conduction of a stimulation artefact from the stimulating electrode is not the source of cortical half harmonic activity. **5) Non-linear response to stimulation:** 1:2 entrainment is also modulated by stimulation parameters [3] (Fig. 1D). Entrainment is lost at higher stimulation amplitudes, demonstrating that we are not simply observing an aliased signal; amplifier saturation might have some effect, but it can be monitored. Additionally, 1:2 entrainment results line up closely with the predictions from the computational model [3].

## Electrical signal chain checklist

When there’s physiological evidence, we can proceed to eliminate the most commonly hypothesised sources of artefacts through benchtop testing and simulations. We begin our checklist with the most difficult to change potential sources, through to the easiest to modulate. **1) Tissue-electrode-interfaces (TEIs):** TEIs have been hypothesised as a potential cause of subharmonic artefacts [7], but can be eliminated through simulations. We simulate the output of 130 Hz monophasic square wave pulses at 130 Hz applied to the resistor network TEI (Fig. 1F, TEI from [7]) and the commonly used TEI of a resistor and capacitor in parallel (Fig. 1E). We did not see subharmonic artefacts induced from either TEI. This is not surprising considering these network models are only made up of linear time-invariant components. A nonlinear element is required to produce anything other than a scaled or phase-shifted output. **2) Stimulator Circuits:** We demonstrate the findings from our characterisation of the Picostim DyNeuMo-2 in a saline tank (Fig. 1H), which shows no subharmonic artefacts for the range of frequencies used. This is in agreement with saline tank results from both the Medtronic Percept PC [8] and the Medtronic Summit RC+S [9] (Fig. 1G). Subharmonic artefacts can arise from stimulators, as demonstrated in the PINS device from residual and structured noise [7]. More researchers should characterise their signal chain (as in [7]) when they observe subharmonic activity of suspected physiological origin instead of ruling responses out as artefacts without further investigation. **3) Analogue-to-digital converters (ADCs):** The ADC is the primary suspect from external signal processing and is easily simulated. We simulate the output of both a conventional downsampled ADC (Fig. 1K) as well as a delta-sigma ADC (Fig. 1L, the more common ADC used in sense-stimulation devices) in response to monophasic square wave pulses of 130 Hz for variable sampling rates. It is possible to observe prominent artefacts at the half harmonic of 130 Hz (Fig. 1I). However, these artefacts occur at precise sampling frequencies and can be predicted (Github ADC Artefact Calculator). Stimulation frequencies of stimulators in use today are already constrained by certain factors [10] and this should be included as another constraint. Looking to the future, manufacturers should characterise and design their electrical signal chain to avoid masking biomarkers.

In summary, through characterisation of the stimulating device and ADC through benchtop testing and simulations in combination with the modulation of biomarkers by state, location and stimulation, we can effectively rule out the presence of subharmonic artefacts. Detailed study of subharmonic entrainment is still limited to a few research groups, and replication of these findings will further increase confidence that subharmonic activity does not have to be artefactual. By better understanding the system that we are stimulating, we may be able to predict neural responses to stimulation, obtain mechanistic insights into therapy and design robust adaptive algorithms.
